# Microglia enhanced the angiogenesis, migration and proliferation of co-cultured RMECs

**DOI:** 10.1186/s12886-018-0886-z

**Published:** 2018-09-17

**Authors:** Xinyi Ding, Ruiping Gu, Meng Zhang, Hui Ren, Qinmeng Shu, Gezhi Xu, Haixiang Wu

**Affiliations:** 1 Department of Ophthalmology, Eye and ENT Hospital of Fudan University, 83 Fen Yang Road, Shanghai, 200031 People’s Republic of China; 2 Institute of Eye Research, Eye and ENT Hospital of Fudan University, Shanghai, China; 3 Key Laboratory of Myopia of State Health Ministry (Fudan University), Shanghai, China; 4 Shanghai Key Laboratory of Visual Impairment and Restoration(Fudan University), Shanghai, China

**Keywords:** Microglia, Retinal microvascular endothelial cells, Angiogenesis, Inflammation, Retinopathy

## Abstract

**Background:**

Attention is increasingly being given to microglia-related inflammation in neovascular diseases, such as diabetic retinopathy and age-related macular disease. Evidence shows that activated microglia contribute to disruption of the blood–retinal barrier, however, the mechanism is unclear. In this study, we aimed to clarify whether and how microglia affect the function of retinal microvascular endothelial cells (RMECs).

**Methods:**

We activated microglia by Lipopolysaccharides (LPS) stimulation. After co-culturing static or activated microglia with RMECs using the Transwell system, we evaluated the function of RMECs. Vascular endothelial growth factor-A (VEGF-A) and platelet-derived growth factor-BB (PDGF-BB) levels in the supernatant from the lower chamber were evaluated by ELISA. Angiogenesis, migration, and proliferation of RMECs were assessed by tube formation, wound healing, and WST-1 assays. The expression levels of tight junction proteins (ZO-1 and occludin) and endothelial markers (CD31 and CD34) were examined by Western blot analysis.

**Results:**

We successfully established an LPS-activated microglia model and co-culture system of static or activated microglia with RMECs. In the co-culture system, we showed that microglia, especially activated microglia stimulated VEGF-A and PDGF-BB expression, enhanced angiogenesis, migration, proliferation, and permeability, and altered the phenotype of co-cultured RMECs.

**Conclusions:**

Microglia, especially activated microglia, play important roles in angiogenesis and maintenance of vascular function hemostasis in the retinal microvasculature. The mechanism needs further investigation and clarification.

## Background

Microglia are important immune cell residents of the central nervous system, as well as the retina [1]. Microglial activation is involved in many important retinopathies, including light-induced photoreceptor degeneration, uveitis, age-related macular degeneration (AMD), and diabetic retinopathy [2–5].

Vascular endothelial cells are key components involved in the blood–retina barrier function and angiogenesis. Increased levels of blood glucose, advanced glycosylation end products (AGEs), and oxidative stress in diabetes drastically alter endothelial cell metabolism and induce endothelial cell dysfunction [6].

Increasing attention has recently been given to microglia-related inflammation in neovascular diseases, both in the central nervous system (CNS) and the retina. For example, it has been reported that diabetic retinopathy is closely related to retinal microvascular system damage, such as breakdown of the blood–retina barrier and angiogenesis and activation of hyperglycemia and/or hypoxia [7]. Recent studies have shown that retinal inflammation also contributes to the pathogenesis of diabetic retinopathy, and that activated microglia are present during the early stages of diabetic retinopathy and cluster in the retinal microvasculature [8–13]. However, the mechanism by which microglia affect retinal microvascular pericytes and endothelial cells needs further clarification. In our previous study, we showed that activated microglia induce production of reactive oxygen species (ROS) and promote apoptosis in co-cultured retinal microvascular pericytes [14]. Thus, in this study, we aimed to clarify whether microglia affect the functions of RMECs such as angiogenesis, migration, and proliferation.

## Methods

### RMECs culture

Rat primary RMECs were purchased from the Cell Biologics Company (catalogue no. RA-6065; Chicago, IL, USA). The cell line was recovered and cultured in accordance with the supplier’s instructions. Cells from passages 4–10 were used in this study.

### Primary retinal microglia culture

Microglia were isolated from the retinas of newborn Sprague–Dawley rats. Newborn Sprague–Dawley rats (1–3 days old) were obtained from the Shanghai SLAC Laboratory Animal Company, and were sacrificed by decapitation. Treatment of animals was complied with the rules of the “Instruction and Administration of Experimental Animals”, and was approved by the Eye and ENT Hospital of Fudan University. The dissected retinas were collected and digested with 0.25 mg/mL trypsin at 37 °C. After 5 min, the trypsin was deactivated with DMEM/F12 (Gibco, Carlsbad, CA, USA) containing 20% FBS (Gibco) and 1% penicillin/streptomycin (Sigma-Aldrich, Billerica, MA, USA). The digested tissues were mechanically dissociated into a single cell suspension and collected by centrifugation. The cells were then resuspended in a DMEM: F12 (1:1) solution containing 20% FBS and plated in T75 cell culture dishes at 10^6^ cells/dish. After 12 days, the supernatant containing microglia was collected and centrifuged, and the cells were resuspended at the appropriate density, depending on the experiment. The microglia were identified by immunocytochemical staining using microglia-specific antibodies, OX42 (targeting CD11b/c) and ED1 (targeting CD68). The morphology of the isolated microglia was examined by phase-contrast microscopy (Nikon) and fluorescence microscopy (Leica Microsystems).

### Flow cytometry

Isolated microglia were collected in DMEM/F12 containing 20% FBS and centrifuged for 10 min. After washing in PBS, the cell precipitate was resuspended in blocking solution (PBS containing 5% FBS and 1% BSA) and labelled with Alexa-Fluor-647-conjugated mouse anti-OX42 (1:100, Abcam, Cambridge, MA) for 15 min at 4 °C. The OX42-positive cells were counted by flow cytometry (Coulter Epics XL, Beckman-Coulter, Fullerton, CA).

### Immunofluorescence assay

For immunofluorescence assays, cell cultures were fixed with 4% paraformaldehyde in 0.01 M PBS for 10 min and then washed with PBS. After washing, the cultures were incubated in blocking buffer (5% normal goat serum) for 30 min at 37 °C. The cells were then incubated overnight at 4 °C with primary antibodies specific for markers of microglia (mouse OX42, 1:100, and mouse ED1, 1:100; Abcam, Cambridge, MA, USA) or RMECs (mouse anti-von Willebrand factor (vWF, 1:100; Abcam). After washing with PBS, the cells were incubated with the appropriate secondary antibodies for 30 min at 37 °C and counterstained with DAPI (1:1000; Molecular Probes/Thermo Fisher Scientific, Waltham, MA, USA). The labelled cells were examined by fluorescence microscopy (Leica Microsystems).

### Activation of microglia by LPS

The harvested microglia were seeded at 10^6^ cells/well in a six-well culture plate pre-coated with 20 μg/mL poly-d-lysine (Sigma-Aldrich). Twenty-four hours after seeding, each well was washed three times with 0.1 M PBS and incubated with culture medium containing 0, 0.1, 1, 10, 100, or 1000 ng/mL lipopolysaccharide (LPS) (*Escherichia coli* OB4:1111; Sigma-Aldrich) for 24 h.

### Assessment of microglial viability

The effects of LPS on the viability of microglia were measured using the cell proliferation reagent water soluble tetrazolium-1 (WST-1; Roche, Basel, Switzerland). The WST-1 assay is based on the cellular reduction of WST-1 by viable cells. Microglia were seeded in a 96-well microplate at 4 × 10^3^ cells/well in 100 μL culture medium containing 0, 0.1, 1, 10, 100, or 1000 ng/mL LPS. The cells were incubated for 48 h at 37 °C in 5% CO_2_, and 10 μL WST-1 reagent was added to each well and incubated for 4 h at 37 °C in 5% CO_2_. The plate was thoroughly shaken for 1 min on a shaker. To detect the production of formazan, the absorbance of each well at 420–480 nm was measured relative to the blank wells on a microplate reader.

### Measurement of microglial cytokine concentrations

After exposure to LPS, the culture media was collected and centrifuged. Aliquots of the supernatant (50 μL) were collected to measure the concentrations of TNFα and IL-1β using enzyme-linked immunosorbent assay (ELISA) kits (R&D Systems, Minneapolis, MN, USA).

### Transwell co-culture of microglia and RMECs

**Fig. 1:**
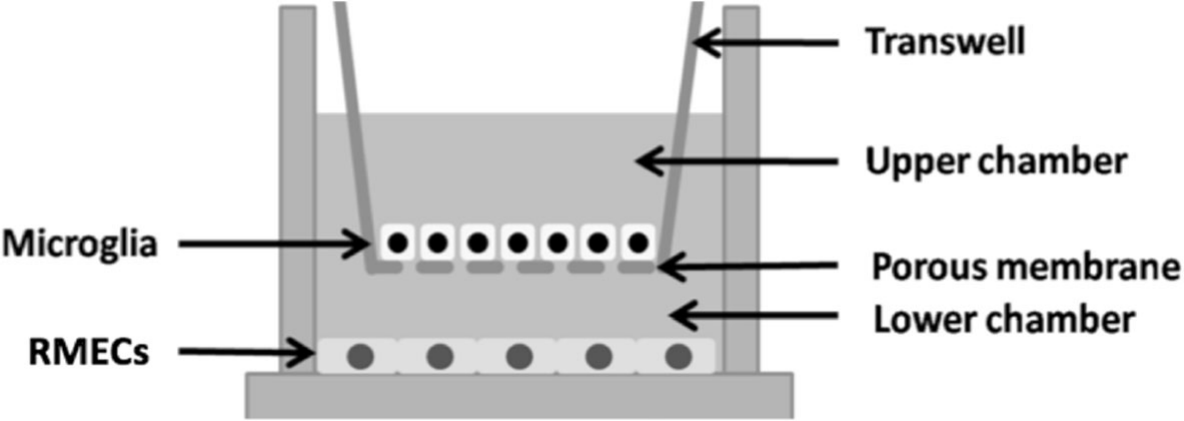
Schematic diagram of the Transwell coculture system. The Transwell system consists of two chambers separated by a porous membrane. The RMECs were placed on the bottom of the lower chamber and the microglia were placed on the membrane of the upper chamber. (Page7, Paragraph 1)

Freshly collected microglia were seeded onto 12-well Transwell collagen-coated membrane inserts (Corning Co., Corning, NY, USA). Separately, RMECs were grown to confluence in a collagen-coated 12-well plate. The microglia and RMECs were incubated in basal media for 24 h before co-culturing, and the Transwell inserts containing the retinal microglia (treated with or without LPS for 24 h) were placed into the wells containing RMECs. The 0.4 μm pore size of the Transwell prevents direct cell–cell interactions but allows the diffusion of soluble factors across the membrane (Fig. 1). After 24 h, the co-cultured cells were separated and cultured in fresh culture mediums for another 24 h, and then, the supernatant and cells were collected for further research. The experiment was divided into three groups: Con (RMECs without microglia), MG: (RMECs with static microglia), LPS-MG (RMECs with activated microglia).

### Levels of angiogenesis-related growth factors in RMECs measured by ELISA

The supernatant of RMECs was collected and subjected to ELISA. The levels of vascular endothelial growth factor-A (VEGF-A) and platelet-derived growth factor-BB (PDGF-BB) were evaluated by sandwich ELISA (human VEGF-A and PDGF-BB ELISA kits, Abcam) according to the manufacturer’s instructions. Colorimetric analysis was performed and the absorbance was measured using an ELISA plate reader.

### Tube formation assays

Tube formation assays were conducted on Matrigel (BD Biosciences, Franklin Lakes, NJ, USA). A 96-well plate was coated with 50 μL/well Matrigel at 37 °C for 30 min. After co-culturing with microglia for 24 h, RMECs were seeded on the Matrigel at 1.5 × 10^4^ cells/well in 100 μL medium. After 4 h, tube formation was observed and photographed with a microscope (Leica Microsystems). Images were analyzed using ImageJ (NIH public domain), and four parameters were measured for quantification of tube formation: total tube length, number of nodes, number of branches, and total branch length.

### Wound healing assays

RMECs were plated at a density of 10^4^ in 24-well plates and grown to 100% confluence. To investigate the migration capability of RMECs, a wound was created by scratching the confluent monolayer down the middle of each well using a 10 μL pipette tip. Scratched RMECs were washed with PBS, and then 500 μL of fresh DMEM without serum was added to each well. Cells were imaged immediately after stimulation (0 h) and then at 6, 12, and 18 h after wounding. ImageJ software was utilized to determine the percentage of wound closure.

### Assessment of RMEC proliferation

RMECs were collected and seeded in 96-well plates at 1 × 10^4^ cells/well and treated with WST-1 for 4 h. The absorbance of the wells was read at 450 nm using the Benchmark Plus microplate reader to evaluate RMEC proliferation.

### Phenotype and tight junction of RMECs

ZO-1 and occludin are markers of tight junctions in RMECs.CD31 and CD34 are markers of endothelial cells. RMECs were harvested, and the protein expression levels of zonula occludens-1(ZO-1), occludin, CD31 and CD34, were determined by Western blotting. After the proteins were transferred onto a PVDF membrane, the blots were incubated with primary antibodies (mouse anti-ZO-1, 1:100; mouse anti-occludin, 1:100; mouse anti-CD31, 1:100; mouse anti-CD34, 1:100; all from Abcam) and probed with a horseradish-peroxidase-conjugated secondary antibody. Protein expression was detected using an enhanced chemiluminescence kit. The density of each band was quantified using ImageQuant software (ImageQuant TL v. 7.0, GE Healthcare, Piscataway, NJ, USA). All samples were assayed in triplicate.

### Statistical analysis

All statistical analyses were performed using GraphPad Prism 5.0. Measurement data were presented as‾x ± s. Differences between groups were evaluated using unpaired one-way ANOVA and LSD post-hoc tests. Each experiment was repeated three times. *P* < 0.05 was considered statistically significant.

## Results

### Characterization of rat retinal microglia and RMECs

**Fig. 2:**
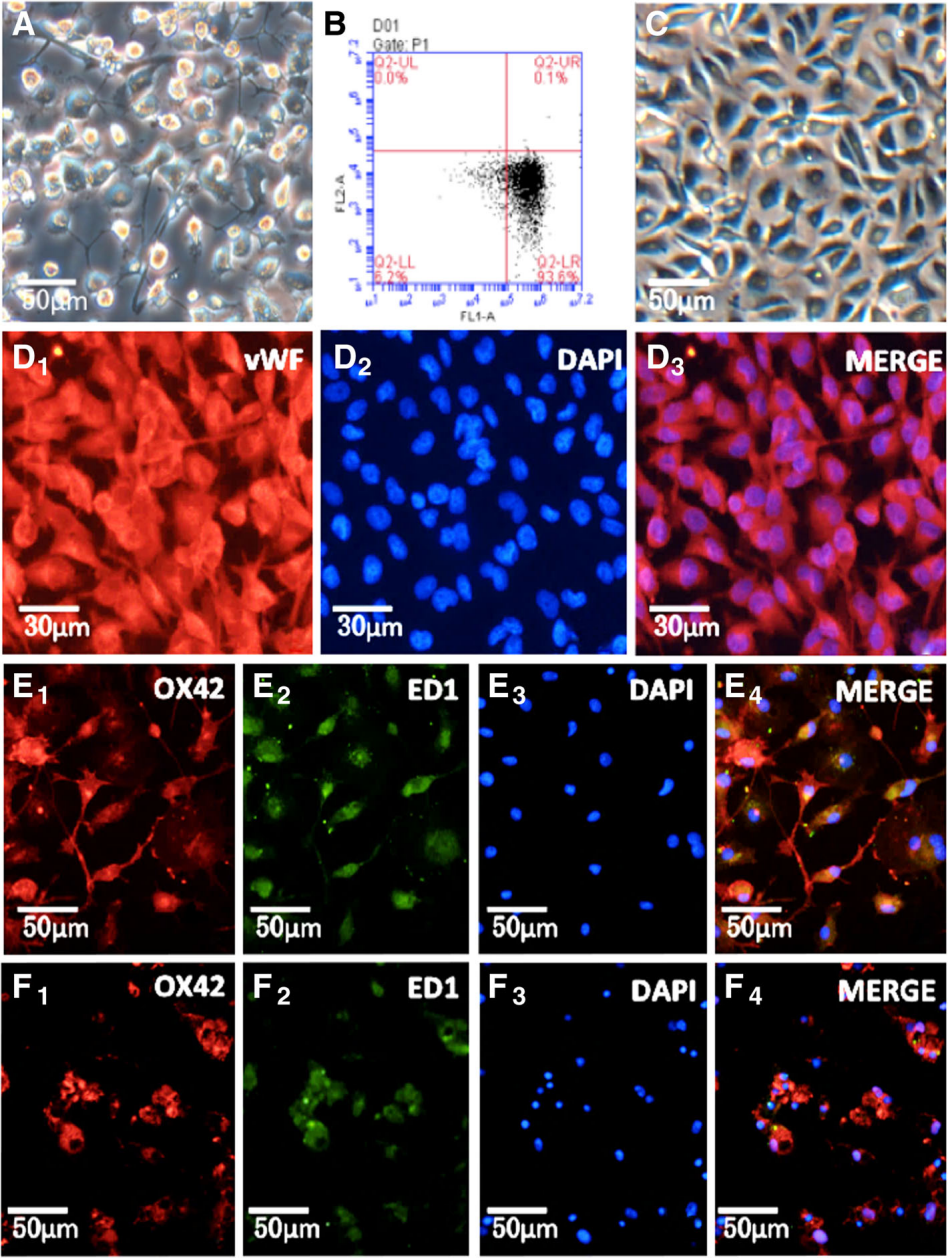
Characterization of rat retinal microglia and RMECS. A: Cultures of primary isolated microglia. B: The purity of isolated microglia detected by low cytometry through its specific surface marker CD11b is 93.6%. C: Culture of RMECs. D1-D3: Immunofluorescent detection of RMECs marked by vWF, DAPI and both. E1–E4: Normal microglia are static. F1 − F4: Exposure to 100 ng/mL LPS for 24 h altered the morphology of the microglia from a ramified state with long processes to the activated state with an amoeboid appearance. E1-E4, F1-F4 has been published in our previous research. Abbreviations: vWF, von-Willebrand factor; LPS, lipopolysaccharide. (Page9–10)

Microglia were harvested from 14-day-old primary mixed glial cultures using the “shaking-off” method. Twenty-four hours after purification and reseeding, the microglia had recovered from the isolation process and resumed their normal morphology of a short, single process and small cell soma (Fig. 2A). The purity of isolated microglia detected by low cytometry through its specific surface marker CD11b, was 93.6% (Fig. 2B). The cultured RMECs had round nuclei and a fusiform shape (Fig. 2C), and vWF was abundantly expressed throughout the RMECs (Fig. 2 D1). Immunofluorescence studies showed cell staining of CD11b/c (by OX42) (Fig. 2E_1_), and CD68 (by ED1) (Fig. 2E_2_).

### Effects of LPS on microglial morphology

To complete our following experiment, we needed to establish a model of activated microglia using LPS. To confirm LPS activation of microglia, the morphology of the microglia was evaluated. The purified microglia presented a static morphology with a branching shape, and immunofluorescence staining confirmed expression of CD11b/c and CD68 in the resting microglia (Fig. 2E_1_–E_4_). After treatment with 100 ng/mL LPS for 24 h, the microglia became rounder and larger, developing a characteristic amoeboid shape consistent with their activation (Fig. 2F_1_–F_4_), as we reported previously [14].

### Effects of LPS on microglial viability

**Fig. 3:**
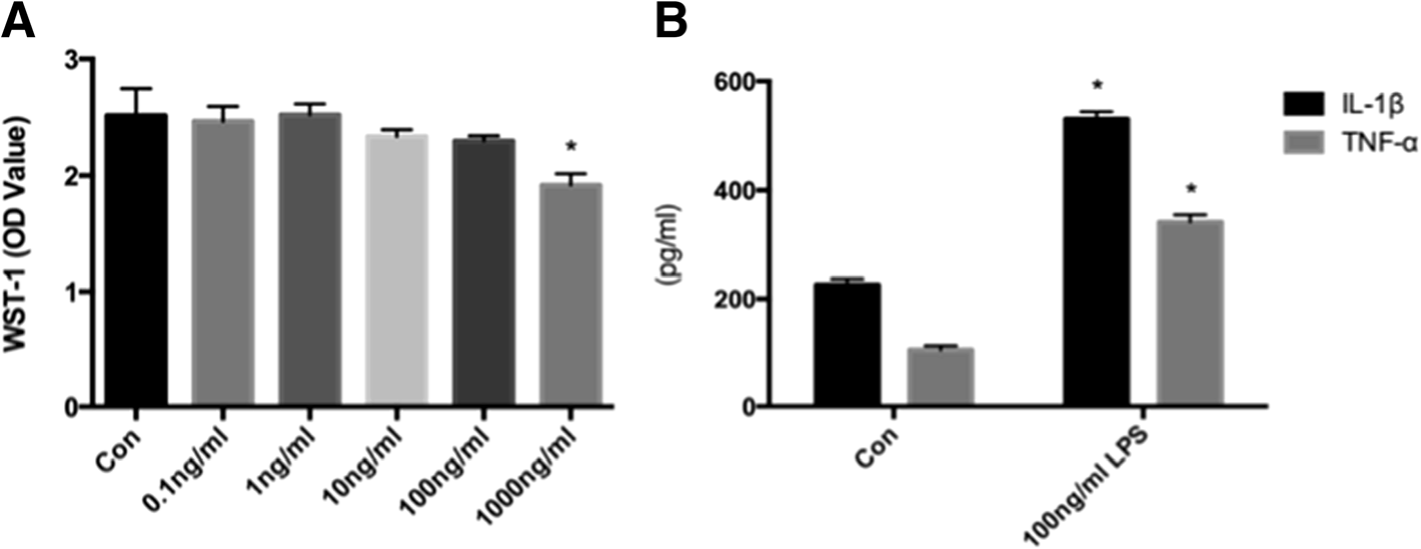
**a** Concentration-dependent effects of LPS on the viability of microglia. Microglia were exposed to LPS (0, 0.1, 1, 10, 100, or 1000 ng/mL) for 24 h. Microglial viability was measured with WST-1 reagent. LPS at 1000 ng/mL significantly reduced the viability of microglia. This part has been published in our previous research. **b** Effects of LPS on TNF and IL-1beta secretion. Microglia were exposed to LPS (0 and 100 ng/mL) for 24 h. The TNFα and IL-1beta concentration in the supernatant was measured using an ELISA kit. LPS at concentrations of 100 ng/mL significantly increased the production of TNF and IL-1beta. Results are means ± SD (*n* = 3 per group). *P < 0.05 vs Con using one-way ANOVA. Abbreviations: Con, microglia without LPS; WST-1, water soluble tetrazolium-1; LPS, lipopolysaccharide; TNFα, tumor necrosis factor; IL-1β, interleukin 1β. (Page 10, paragraph3–4)

To confirm that LPS had no cytotoxic effect on the activated microglia, cell viability was evaluated using WST-1 reagent. At concentrations of 0.1–100 ng/mL, LPS did not significantly reduce cell viability. However, at 1000 ng/mL, LPS significantly reduced the viability of the microglia, indicating that 1000 ng/mL LPS has a cytotoxic effect on the microglia (Fig. 3a), as we reported previously [14]. Thus, we chose 100 ng/mL LPS to activate microglia, avoiding its cytotoxic effect on cells.

### Effects of LPS on TNFα and IL-1β secretion from microglia

To confirm LPS activation of microglia, the pro-inflammatory factors of the microglia were evaluated. As shown in Fig. 3, TNFα and IL-1β were significantly overexpressed after adding 100 ng/mL LPS to the microglial culture medium. The TNFα and IL-1β levels were 3.22 ± 0.14 and 2.35 ± 0.08 times higher, respectively, than the levels in unstimulated microglia after 24 h LPS activation (Fig. 3b). Activation of microglia by LPS was shown by microglia morphology change and increased TNFα and IL-1β secretion.

### Effects of static or activated microglia on the expression of angiogenesis-related factors in RMECs

After successfully establishing activated model of microglia, we co-cultured pretreated microglia with RMECs in the Transwell system to see the effects of microglia on RMECs. Freshly collected microglia were seeded onto 12-well Transwell collagen-coated membrane inserts (Corning Co., Corning, NY, USA) (treated with or without LPS for 24 h). Separately, RMECs were grown to confluence in a collagen-coated 12-well plate for 24 h. After co-culture for 24 h, as shown in Fig. 1, the cells were separated and cultured in fresh culture mediums for another 24 h, then then, the supernatant and cells were collected for further research. The experiment is divided into three groups: Con (RMECs without microglia), MG: (RMECs with static microglia), LPS-MG (RMECs with activated microglia).

**Fig. 4:**
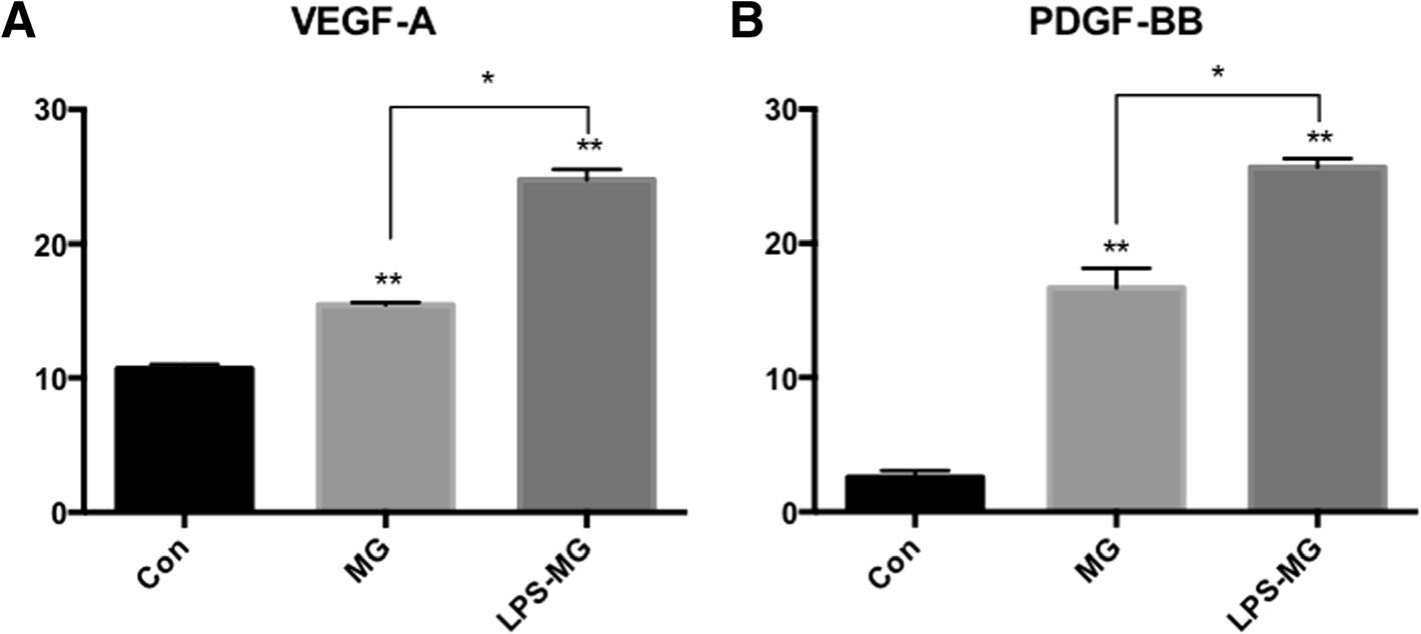
Effects of static or activated microglia on angiogenesis relative factors expression of RMECs. **a** VEGF-A. **b** PDGF-BB. Compared with control RMECs, microglia significantly increased expression and release of VEGF-A and PDGF-BB in RMECs. LPS activated microglia enhanced the effects of static microglia to RMECs. Each bar graph indicates means ± SD of three independent experiments. *Significant difference in results between the two compared groups. *P < 0.05 using one-way ANOVA. **P < 0.01 using one-way ANOVA. Abbreviations: Con, control RMECs (i.e., cultured without microglia); MG, REMCs co-cultured with static microglia; LPS-MG, REMCs co-cultured with activated microglia; LPS, lipopolysaccharide; VEGF-A, Vascular endothelial growth factor-A; PDGF-BB, Platelet-derived growth factor-BB. (Page 11, paragraph 2)

The effects of microglia on the expression of angiogenesis-related factors in RMECs were evaluated by ELISA using the supernatant collected from the lower chamber. In the co-culture system, microglia significantly increased the release of VEGF-A and PDGF-BB from RMECs, compared to the RMECs without microglia. Furthermore, LPS-activated microglia enhanced the effects of static microglia on RMECs (Fig. 4). We conclude that microglia, especially activated microglia induced VEGF-A and PDGF-BB secretion of RMECs.

### Effect of static or activated microglia on RMECs tube formation

**Fig. 5:**
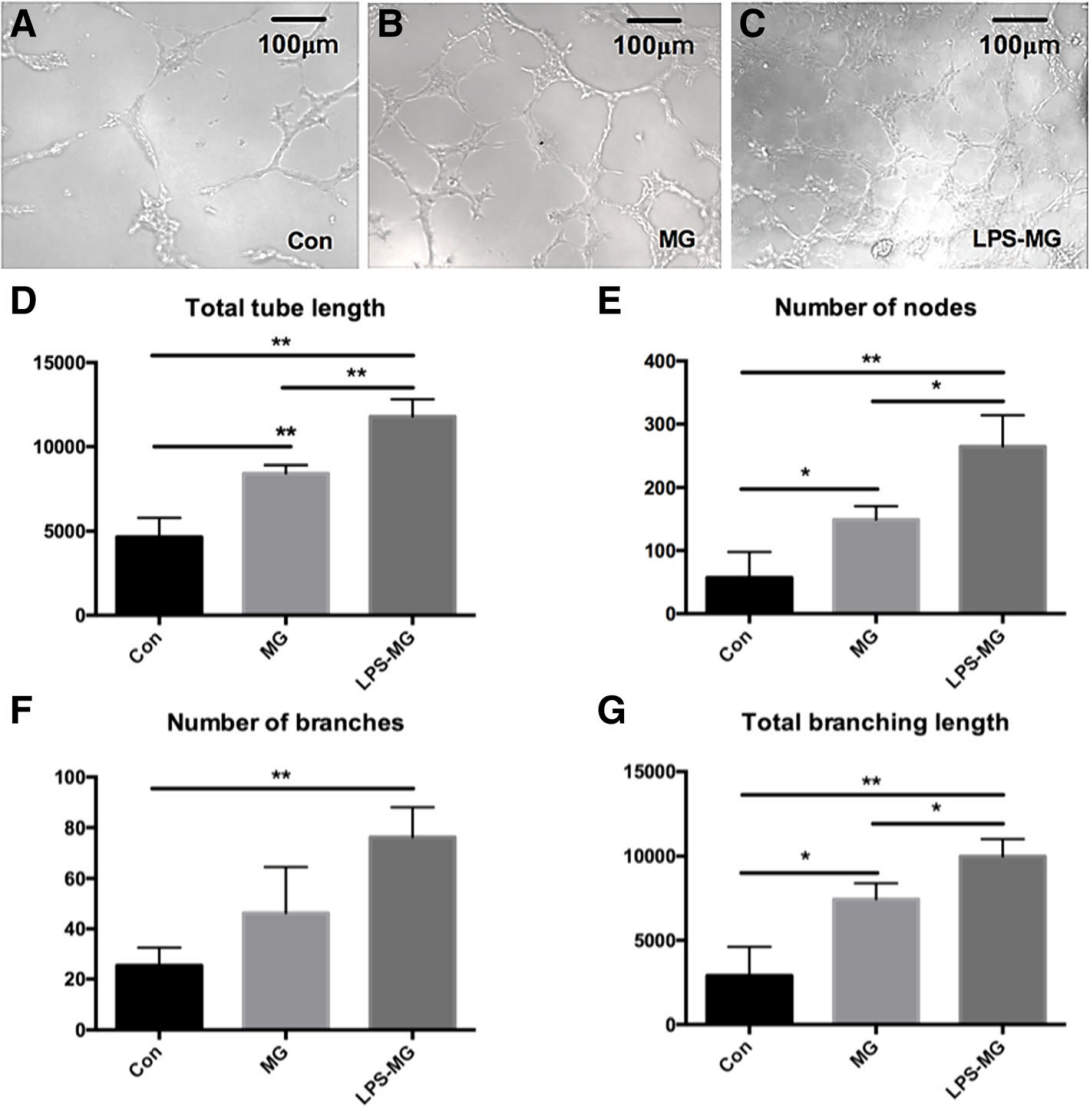
Effects of static or activated microglia on tube formation of RMECs. **a-c** One representative experiment of tube formation of three different groups at 4 h. **d-g** Total tube length, number of nodes, number of branches, total branching length quantified by ImageJ. Both static and LPS-activated microglia stimulated tube formation in the RMECs. In addition, the effects on RMEC tube formation were enhanced by the activated microglia compared with the static microglia. Each bar graph indicates means ± SD of three independent experiments. Significant difference in results between the two compared groups. *P < 0.05 using one-way ANOVA. **P < 0.01 using one-way ANOVA. Abbreviations: Con, control RMECs (i.e., cultured without microglia); MG, REMCs co-cultured with static microglia; LPS-MG, REMCs co-cultured with activated microglia; LPS, lipopolysaccharide. (Page 11, paragraph 3)

Preprocessing and grouping of cells were as mentioned above. The effect of microglia on the angiogenesis ability of RMECs was measured with the tube formation assay, an important in vitro model for angiogenesis. As shown in Fig. 5, both static and activated microglia stimulated tube formation in the RMECs. In addition, the effects on RMECs tube formation were enhanced by the activated microglia compared with the static microglia (Fig. 5). The tube formation assay suggested that microglia, especially activated microglia induced the angiogenic abilities of RMECs.

### Effect of static or activated microglia on RMECs migration

**Fig. 6:**
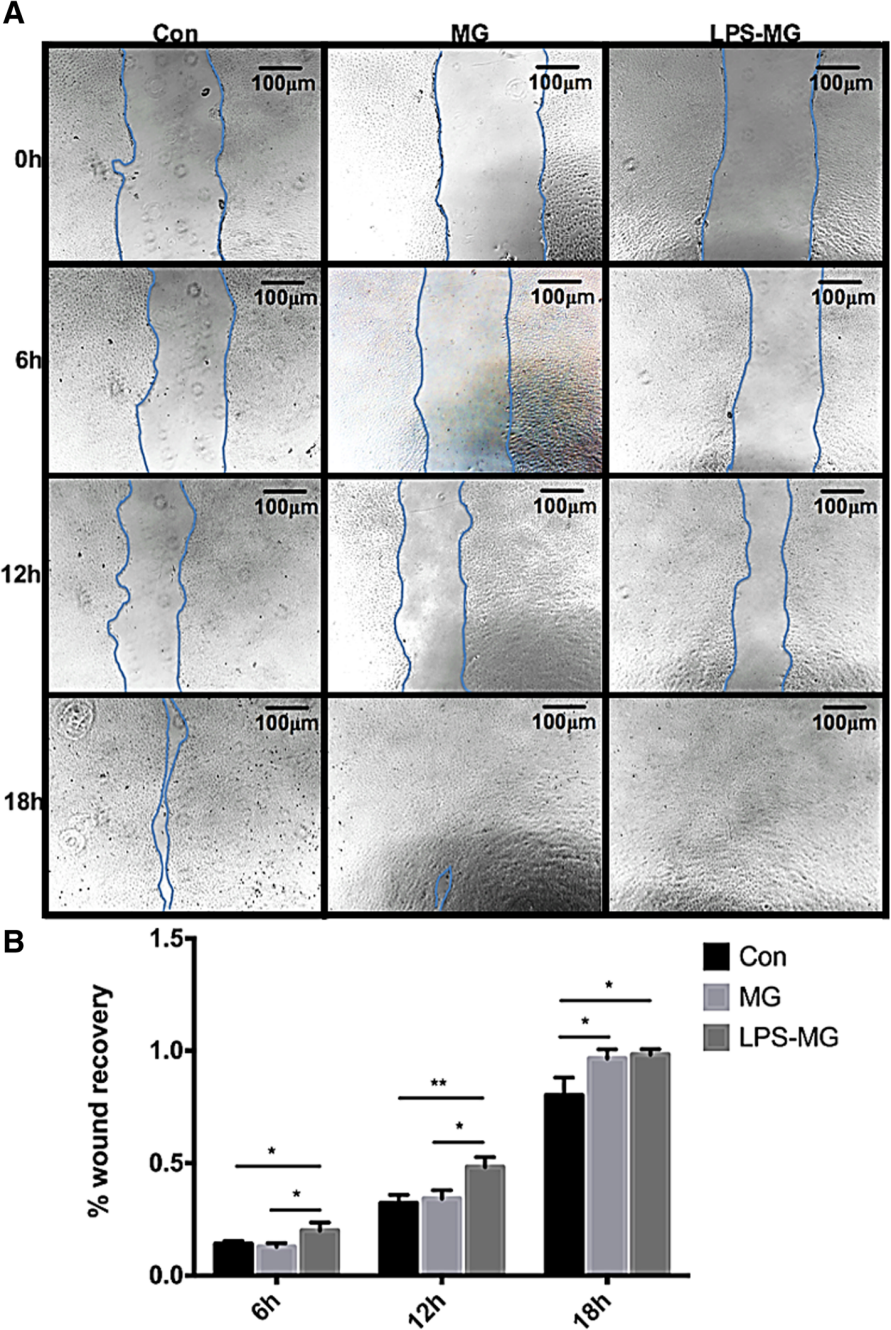
Effects of static or activated microglia on migration of RMECs. **a** One representative experiment of three different groups at 0 h, 6 h, 12 h, 18 h. **b** Wound recovery percentage analyzed by ImageJ. We see static microglia significantly stimulated wound recovery at 18 h, at the same time, activated microglia significantly stimulated wound recovery at 6 h, 12 h and 18 h. Each bar graph indicates means ± SD of three independent experiments. *Significant difference in results between the two compared groups. *P < 0.05 using one-way ANOVA. **P < 0.01 using one-way ANOVA. Abbreviations: Con, control RMECs (i.e., cultured without microglia); MG, REMCs co-cultured with static microglia; LPS-MG, REMCs co-cultured with activated microglia; LPS, lipopolysaccharide. (Page 11, paragraph 4)

Preprocessing and grouping of cells were as mentioned above. The effect of microglia on RMEC migration was measured with the wound healing assay. As shown in Fig. 6, both static and activated microglia induced RMEC migration. Static microglia significantly stimulated wound recovery at 18 h, while activated microglia significantly stimulated wound recovery at 6, 12, and 18 h (Fig. 6). The wound healing assay revealed that microglia, especially activated microglia increased the migration of RMECs.

### Effect of static or activated microglia on RMEC proliferation

**Fig. 7:**
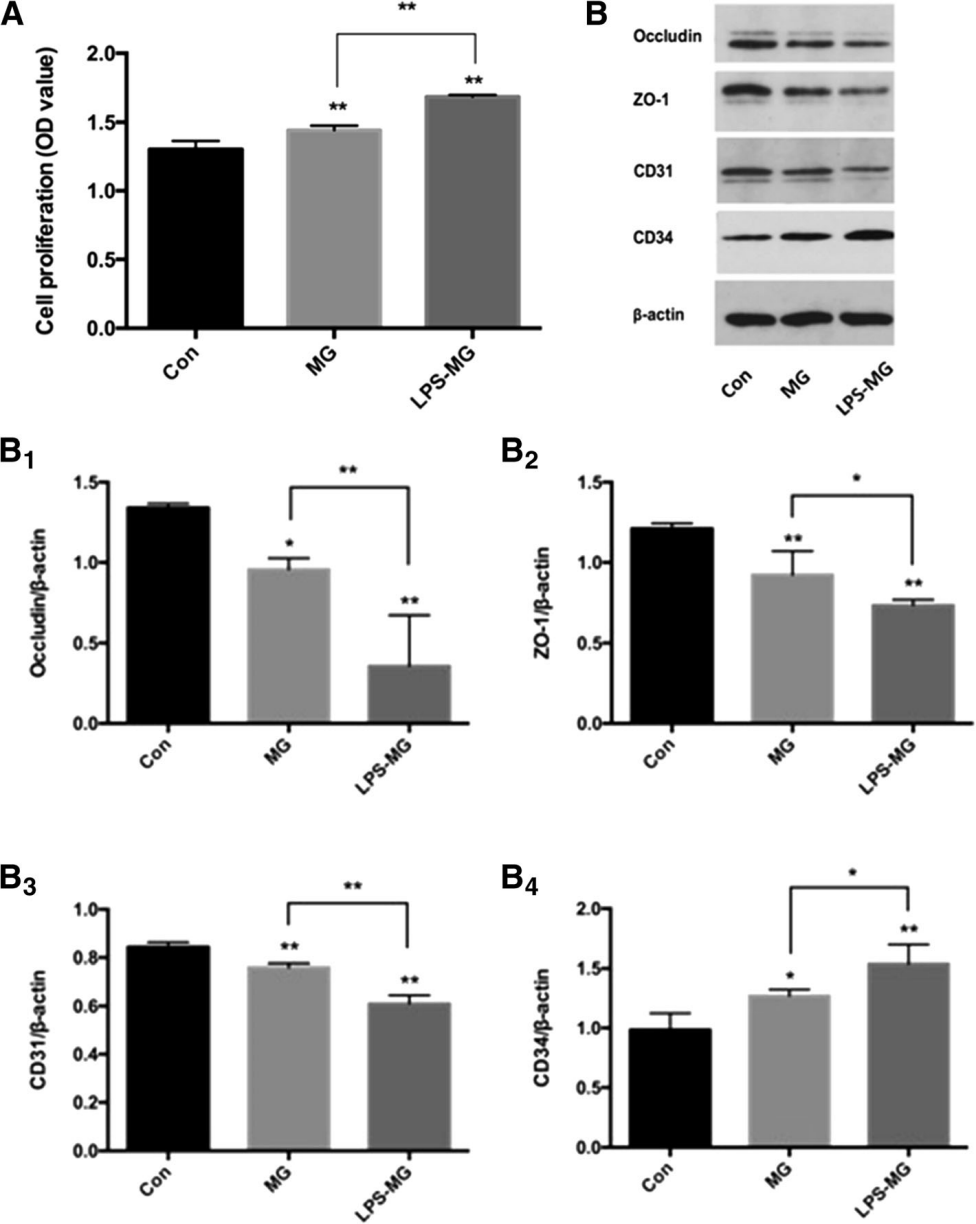
A. Effects of static or activated microglia on proliferation of RMECs. RMECs were cocultured with microglia (with or without LPS) for 24 h, WST-1 reagent was used to evaluate RMECs proliferation ability. We found that both static and activated microglia induced RMECs proliferation, and LPS further enhanced the stimulatory effect of microglia on RMECs. B. Effects of static or activated microglia on permeability and phenotype of RMECs. For evaluation of permeability and phenotype of RMECs, after cocultured with microglia (with or without LPS) for 24 h, we collected RMECs and tested protein expression of occludin, ZO-1, CD-31, and CD-34. Both static and activated microglia significantly reduced the expression of occludin and ZO- 1, markers of cell permeability, in RMECs compared with RMECs without microglia. Regarding phenotype, both static and activated microglia reduced CD31 and increased CD34 expression, which are endothelial markers, in RMECs (B). *P < 0.05 using one-way ANOVA. **P < 0.01 using one-way ANOVA. Abbreviations: Con, control RMECs (i.e., cultured without microglia); MG, REMCs co-cultured with static microglia; LPS-MG, REMCs co-cultured with activated microglia; LPS, lipopolysaccharide; ZO-1, zonula occludens-1. (Page 12, paragraph 2–3)

Preprocessing and grouping of cells were as mentioned above. The effect of microglia on RMECs proliferation was measured with WST-1 reagent. We found that both static and activated microglia induced RMEC proliferation, and LPS further enhanced the stimulatory effect of microglia on RMECs (Fig. 7a). The WST-1 assay showed that microglia, especially activated microglia promoted migration of RMECs.

### Effects of static or activated microglia on tight junctions and the phenotype of RMECs

Preprocessing and grouping of cells were as mentioned above. For evaluation of the tight junction and phenotype of RMECs, protein levels of occludin, ZO-1, CD-31, and CD-34 were measured with Western blotting. Both static and activated microglia significantly reduced the expression of tight junction occludin and ZO-1, markers of cell permeability, in RMECs compared with control cells. Regarding phenotype, both static and activated microglia significantly reduced CD31 and increased CD34 expression, which are endothelial markers, in RMECs (Fig. 7b). We concluded from these data that microglia, especially activated microglia, destroyed the tight junction of RMECs, which may contribute to the increased permeability of vasculature. In addition, microglia, especially activated microglia changed phenotype of RMECs, and the specific effect of these changes needs further investigation.

## Discussion

The retinal microvasculature is composed of endothelial cells and pericytes. Diabetic retinopathy is first considered to be a simple microangiopathy. In the early stages of diabetic retinopathy, endothelial cell proliferation and pericytes apoptosis leads to damage of the blood-retinal barrier (BRB) [15].

Recently, new insights into retinal physiology have led to the emergence of the concept of the retinal neurovascular unit, [16–18] which is composed of retinal neurons (photoreceptors, horizontal, etc), their supporting cells (astrocytes and Müller glial cells), and the vascular beds (endothelial cells and pericytes). In diabetic retinopathy, metabolic alterations may first lead to glial dysfunction, then induces inflammation, and neuronal apoptosis. Neurodegeneration also contributes to the breakdown of the blood–retinal barrier (BRB) [16, 17].

In addition, the role of microglia in diabetic retinopathy is currently of particular interest. However, the mechanisms by which the microglia affect the retinal microvascular pericytes and endothelial cells in diabetic retinopathy needs further clarification.

Combining our previous study with the present study, we showed that activated microglia increased oxidative stress damage and promoted apoptosis in pericytes; [14] and induced VEGF production, angiogenesis, migration, and proliferation. Activated microglia also destroyed the tight junction of endothelial cells affecting the integrity of the microvasculature, leading to BRB breakdown and contributing to neovascularization.

As the most important immune monitoring cell in the retina, microglia may manifest as static or activated states. It has been documented that microglia cells are abnormally activated in a variety of retinal diseases,, including diabetic retinopathy [8, 19]. LPS stimulation is the most commonly used and effective method to activate microglia in vitro [20–22]. LPS could induce NF-κB activation and inflammatory cytokines release [23]. We initially used 100 ng/ml of LPS to successfully establish an activated model of microglia, which was proved by an increased expression of the pro-inflammatory cytokines TNFα and IL-1β and the typical morphological changes in the cells. In our study, we co-cultured static or activated microglia with RMECs using the Transwell co-culture system. After 24 h of co-culturing, we collected the RMECs to evaluate their functions. VEGF is a key regulator among growth factors involved in angiogenesis [24]. VEGF-A is the most prevalent member of the VEGF family [25]. In addition to VEGF, PDGF is another well-studied angiogenic growth factor [26, 27]. PDGF-BB is the predominant isoform of the PDGF family expressed in the eye [28]. VEGF and PDGF have a synergic effect on vascular homeostasis [29, 30]. In the second part of our study, we found that microglia stimulate VEGF-A and PDGF-BB expression in, and secretion from, RMECs, with an even stronger effect induced by activated microglia. As shown in the first part of the study, static microglia secreted basal levels of TNFα and IL-1β, which were markedly increased after activation by LPS. Thus, we hypothesize that TNFα and IL-1β function to stimulate VEGF-A and PDGF-BB, as suggested by previous studies [31, 32]. TNFα is able to increase the expression of VEGF, [33] stimulate NF-κB, JNK and p38 signaling pathways, [34, 35] all of which contribute to angiogenic activity [36]. IL-1β induced VEGF production through an src-dependent pathway and MAPK/ERK signal pathways, and both of VEGF and IL-1beta up-regulated vascular angiogenesis and permeability [37–39].

Angiogenesis, migration, and proliferation are the most representative indices of endothelial cell function. The angiogenic growth factors VEGF and PDGF can enhance endothelial angiogenesis, migration, and the proliferation [30, 39–41]. We showed that microglia promoted tube formation, wound healing, and proliferation of co-cultured RMECs, and activated microglia significantly enhanced these effects, in accordance with increased VEGF and PDGF levels.

Hyperpermeability is an important change associated with vascular dysfunction in neovascular diseases [42]. The paracellular permeability of the endothelium depends on the integrity of protein complexes involved in the inter-endothelial junctions [43]. ZO-1 and occludin are indispensable components of tight junctions regulated by pro-inflammatory cytokines and growth factors [44–49]. We showed that microglia downregulated ZO-1 and occludin expression, in agreement with previous studies [50–52]. The inhibitory effects of activated microglia on tight junction proteins were stronger than those of static microglia.

CD31 and CD34 are specific markers of endothelial cells. CD31 (also known as platelet/endothelial cell adhesion molecule-1) is a transmembrane protein that is strongly expressed at cell borders and plays a role in supporting the integrity of endothelial cell–cell junctions [53–55]. CD34, a marker of angiogenesis, is a single-chain transmembrane glycoprotein expressed on the surfaces of hematopoietic precursor cells and capillary endothelial cells [56–58]. In this study, we found that microglia downregulated the expression of CD31 but upregulated the expression of CD34 in co-cultured endothelial cells, and activation of the microglia enhanced these effects. This result is additional evidence illustrating the effects of microglia on tight junction proteins and angiogenesis of endothelial cells.

Vascular endothelial cells and microglia interact in direct or indirect ways. In this study, we used the Transwell system to study their indirect contact mainly through evaluating the release of soluble cytokines. Similar approaches have been evaluated in the CNS system. Endothelial angiogenesis and blood-brain barrier (BBB) dysfunction were caused by the soluble tumor necrosis factor α (TNF-α) released from microglial cells [59, 60]. Circulating micro-vesicles containing miR-27a obtained from LPS-stimulated microglia supernatant damaged the tight junction of RMECs under the OGD condition [61]. In addition, the literature shows that microglia and endothelial cells can also directly interact through the interaction of CD200/CD200R and CX3CL1/CX3CR1, by which endothelial cells are able to regulate the function of microglia [62, 63]. The mechanism and pathways need further investigation.

## Conclusion

In this study, we demonstrated that co-culturing RMECs with microglia resulted in upregulation of VEGF-A and PDGF-BB expression, angiogenesis, migration, and proliferation and downregulated the expression of tight junction proteins in RMECs, and these effects were significantly enhanced by microglial activation. The mechanisms underlying the effects of microglia on the function of adjacent endothelial cells, including the cytokines/proteins and specific pathways involved, need further clarification.

## Abbreviations

IL-1β: Interleukin 1β
LPS: Lipopolysaccharide
MG: Microglia
PDGF-BB: Platelet-derived growth factor-BB
RMECs: Retinal microvascular endothelial cells
TNFα: Tumor necrosis factor
VEGF-A: Vascular endothelial growth factor-A
vWF: von-Willebrand factor
WST-1: Water soluble tetrazolium-1
ZO-1: Zonula occludens-1
